# Primary plasmablastic plasma cell leukemia: A diagnostic conundrum

**DOI:** 10.1002/jha2.1085

**Published:** 2025-01-06

**Authors:** Radu Chiriac, Juliette Fontaine

**Affiliations:** ^1^ Hematology Laboratory Hospices Civils de Lyon, Centre Hospitalier Lyon Sud Lyon France; ^2^ Department of Pathology Hospices Civils de Lyon, Centre Hospitalier Lyon Sud Lyon France

**Keywords:** morphology, myeloma, plasma cell, plasmablastic myeloma

1

A man in his 60s presented with a fever (39.9°C), persistent fatigue, and abnormal weight loss. Laboratory investigations revealed hypercalcemia (3.7 mmol/L), elevated LDH (880 U/L), elevated serum creatinine (2.8 mg/dL), along with anemia (67 g/L), thrombocytopenia (50 × 10^9^/L), and 38% of circulating atypical‐appearing lymphoid cells, varying in size and morphology, with inconspicuous nucleoli, cytoplasmic projections, and basophilic cytoplasm (Figure 1A). The absence of microscopic features characteristic of mature plasma cells hindered precise identification.

**FIGURE 1:**
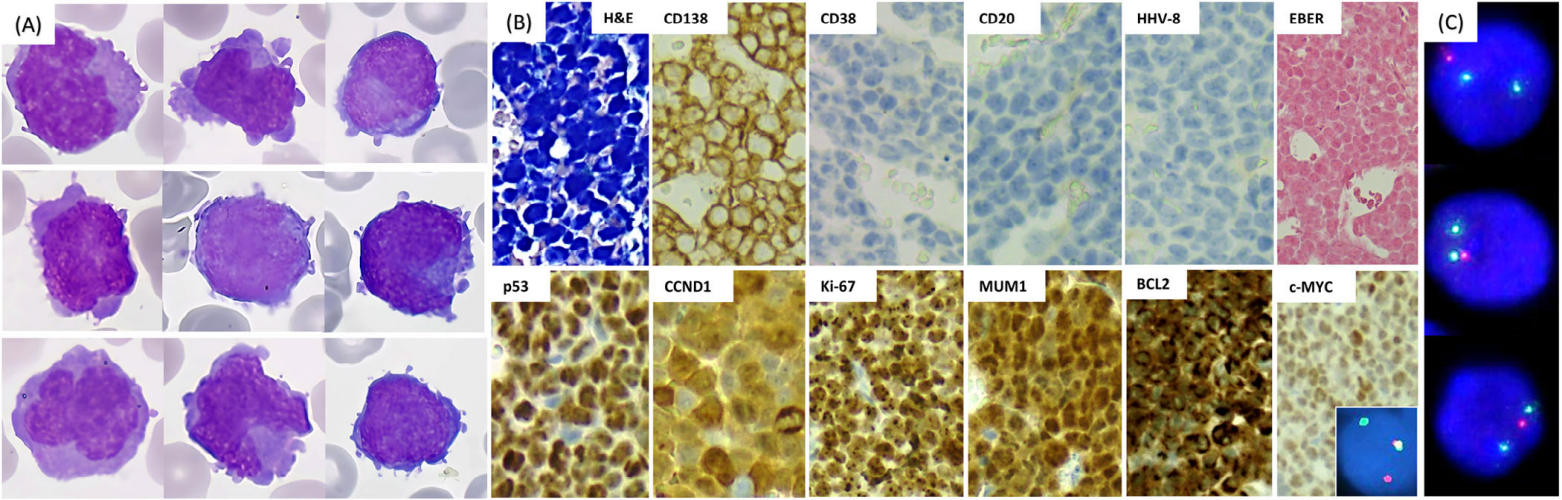
Panel A: May‐Grunwald Giemsa stain, ×100 objective, showing circulating atypical plasma cells. Panel B: Bone marrow core biopsy, ×20 objective, showing neoplastic cells positive for CD138, MUM1, CCND1, BCL2, and c‐MYC, but negative for CD20, CD38, and Human Herpesvirus‐8 (HHV‐8). The Ki‐67 index was nearly 100%, with overexpression of p53 and c‐MYC, and Epstein‐Barr virus (EBV)‐encoded RNA was negative; *MYC* rearrangement (inset). Panel C: Fluorescence in situ hybridization (FISH) analysis using a TP53/NF1 deletion probe – aberrant cell: two green and one orange signal resulting from loss of one orange signal.

Bone marrow core biopsy showed massive infiltration by monomorphic, medium‐sized cells with large, irregular nuclei and scant basophilic cytoplasm. The sample also exhibited a characteristic “starry‐sky” appearance. Immunohistochemical staining (Figure 1B) revealed that the neoplastic cells were positive for CD138, MUM1, CCND1, BCL2, c‐MYC, and kappa light chain, but negative for CD19, CD20, CD38, CD56, BCL6, ALK, and Human Herpesvirus‐8 (HHV‐8). The Ki‐67 proliferation index was nearly 100%, with overexpression of p53 and c‐MYC, and a confirmed *MYC* rearrangement (Figure 1B, inset). In situ hybridization for Epstein‐Barr virus (EBV)‐encoded RNA was negative. Additionally, fluorescence in situ hybridization analysis of enriched plasma cells demonstrated a del(17)(p13.1) involving *TP53* in 95% of cells (Figure 1C). HIV serology was negative. Serum protein electrophoresis showed no M spike but indicated hypogammaglobulinemia. The serum kappa/lambda free light chain ratio was 33.3. magnetic resonance imaging findings showed diffuse degenerative changes in the lumbar spine without vertebral collapse.

Given the phenotype (loss of pan‐B‐cell markers and expression of plasmacytic differentiation markers), bone lesions, absence of EBV and HHV‐8 involvement, and myeloma‐related secondary cytogenetic abnormalities (17p deletion and *MYC* translocation), the diagnosis was more consistent with plasmablastic myeloma (PBM).

An autologous stem cell transplant was planned, but after the third cycle of Dara‐VRd induction, the patient tested positive for severe acute respiratory syndrome coronavirus 2. Two weeks later, he was transferred to the ICU for acute respiratory distress syndrome and, a month later, died from respiratory complications due to multidrug‐resistant *Pseudomonas aeruginosa* ventilator‐associated pneumonia.

The case demonstrates primary plasma cell leukemia with an uncommon plasmablastic morphology. The clinical presentation resembles that of acute leukemia and contrasts with classic plasma cell myeloma. The diverse morphologies of plasma cell neoplasms often mimic other hematopoietic and non‐hematopoietic tumors, leading to their characterization as the “great imitator” [1].

Differential diagnostic considerations for PBM include lymphoid neoplasms with plasmablastic, immunoblastic, or plasmacytoid features, such as diffuse large B‐cell lymphoma (DLBCL), HHV‐8 DLBCL‐NOS, ALK‐positive large B‐cell lymphoma, primary effusion lymphoma, and plasmablastic lymphoma (PBL) [2].

PBM and PBL may present identical morphological and immunophenotypic features, making distinction challenging in rare cases; however, accurate differentiation is crucial for selecting the appropriate therapy and improving prognosis.

PBM, as a plasma cell neoplasm, typically requires treatment with agents targeting plasma cell malignancies. Given the poor prognosis of PBL, no standard of care exists, and CHOP is considered insufficient. Current guidelines recommend more intensive regimens, such as EPOCH, CODOX‐M/IVAC, or hyper‐CVAD. CD38 is a common marker for both diseases and supporting the use of daratumumab‐based regimens, especially when distinguishing between the two is challenging [2].

## AUTHOR CONTRIBUTIONS

Radu Chiriac and Juliette Fontaine wrote the manuscript and conducted the morphological studies. All authors contributed to the final version of the manuscript.

## CONFLICT OF INTEREST STATEMENT

The authors declare no conflict of interest.

## FUNDING INFORMATION

The authors did not receive support from any organization for the submitted work.

## ETHICS STATEMENT

This manuscript respects the ethics policy of CHU Lyon for the treatment of human research participants.

## PATIENT CONSENT STATEMENT

No patient‐identifying data were used. The authors did not obtain written informed consent from the patient but the patient did not object to his data being used for research purposes (as required by the ethics policy of CHU Lyon).

## CLINICAL TRIAL REGISTRATION

The authors have confirmed clinical trial registration is not needed for this submission.

## Data Availability

Data sharing is not applicable to this article as no new data were created or analyzed in this study.
